# Circular RNA mitochondrial translation optimization 1 homologue (CircMTO1) induced by zinc finger protein 460 (ZNF460) promotes oral squamous cell carcinoma progression through the microRNA miR-320a / alpha thalassemia/mental retardation, X-linked (ATRX) axis

**DOI:** 10.1080/21655979.2021.1997699

**Published:** 2021-12-07

**Authors:** Chen Zou, Xia Li, Xiaozhi Lv, Siyuan Wu, Jing Song, Zhe Tang, Hailing Luo, Haigang Wei, Yilong Ai

**Affiliations:** a Foshan Stomatological Hospital, School of Medicine, Foshan University, Foshan, Guangdong, China; b Department of Oral and Maxillofacial Surgery, NanFang Hospital, Southern Medical University, Guangzhou, China

**Keywords:** Circular rna, circMTO1, miR-320a, atrx, znf460, oral squamous cell carcinoma

## Abstract

Oral squamous cell carcinoma (OSCC) is one of the most common cancer types of head and neck cancer, accounting for 95% of all cases. However, the mechanisms underlying the pathogenesis of OSCC remain unclear. Circular RNA (CircRNA) has been extensively studied in the past decades and is a promising direction for the development of OSCC therapeutic targets. In this study, we aimed to investigate the role of circMTO1 in OSCC progression. First, we validated the characterization and expression of circMTO1 in OSCC. It was found that circMTO1 was upregulated in OSCC tumor tissues and cells. Subsequently, we conducted biological experiments. It was found that circMTO1 knockdown inhibited OSCC cell proliferation, migration, and invasion. Furthermore, we conducted a series of experiments to elucidate the underlying mechanisms. A novel circMTO1/miR-320a/ATRX axis was identified. Our results suggest that circMTO1 modulates ATRX expression to accelerate OSCC progression by sponging miR-320a. Moreover, we found that circMTO1 expression in OSCC was transcriptionally regulated by Zinc Finger Protein 460 (ZNF460). Our study showed a novel ZNF460/circMTO1/miR-320a/ATRX signaling in OSCC development.

## Introduction

Oral squamous cell carcinoma (OSCC) is one of the most common cancer types of head and neck cancer, accounting for 95% of all cases. OSCC is also one of the deadliest cancer types worldwide due to its highly metastatic and invasive features [1–3]. Although recent advances in OSCC clinical management have been made, the morbidity and mortality of OSCC are still growing [4,5]. However, the pathogenesis of OSCC remains poorly understood. Therefore, a novel therapeutic strategy for OSCC is urgently needed.

Circular RNA (circRNA) is a class of non-coding RNAs and is characterized by its closed-loop structure [6]. In comparison with its linear form, circRNA has been recognized for its abundant, stable, and conserved expression features in eukaryotic cells [7]. In the past decades, with the innovation of high-throughput technology, several studies have focused on the biological functions of circRNAs in various physiological and pathological processes. Furthermore, emerging evidence suggests that circRNAs are dysregulated and involved in multiple cancer progression [8], including OSCC [9,10]. Zhao W et al. demonstrated that circRNA circUHRF1 modulates OSCC progression through a feedback loop [11]. Liu J et al. elucidated a novel circIGHG/miR-142-5p/IGF2BP3 signaling in OSCC development [12]. Gao L et al. revealed that circCDR1as regulates the autophagy phenomenon in OSCC cells [13]. Published research indicates that circRNAs play an essential role in OSCC progression and could be a promising strategy for developing novel OSCC therapeutic targets.

It has been reported that the circ-mitochondrial translation optimization 1 homologue (MTO1; hsa_circRNA_0007874/hsa_circRNA_104135) plays an important role in cancer cells. CircMTO1 acts as a sponge of miRNA-9 to suppress hepatocellular carcinoma progression [14]. CircMTO1 regulates the progression of cervical cancer by acting as a sponge for miR-6893 [15]. CircRNA circMTO1 suppresses bladder cancer metastasis by sponging miR-221 and inhibiting epithelial-to-mesenchymal transition [16]. However, the role of circMTO1 in OSCC has not been elucidated.

In this study, we hypothesized that circMTO1 plays functional role in the progression of OSCC. Our study investigated the role of circMTO1 in OSCC and found that circMTO1 knockdown inhibits cancer progression by protecting Alpha Thalassemia/Mental Retardation, X-linked (ATRX) from miR-320a regulation in OSCC cells. In conclusion, our findings demonstrate the key function and mechanism of ZNF460/circMTO1/miR-320a/ATRX signaling in OSCC cell progression.

## Materials and methods

### Cell culture and tissue samples

For the OSCC tissues (five cases of tumor tissues and its adjacent normal tissues) used in the current study were collected from NanFang Hospital, Southern Medical University between 2020/06 to 2021/08. Tissues were immediately stored at −80°C for further study. All tissues were identified by two pathologists respectively. To investigate the biological functions of circMTO1 in OSCC, we obtained normal human oral keratinocytes (HOK) cells and OSCC cell lines (SCC-9, SCC-15, CAL-27, HSC-3, and HSC-6) from the ATCC (American Type Culture Collection). DMEM (Gibco, USA) supplemented with 10% fetal bovine serum (FBS) (Gibco) was used as the culture medium for HOK, CAL-27, and HSC-3 cells. DMEM:F12 supplemented with 10% FBS (Gibco) was used as the culture medium for SCC-9 and SCC-15 cells. The cells were cultured for 48 ~ 72 h for each passage. Specific small interfering RNAs (siRNAs) targeting circMTO1, GATA2, MIXL1, GBX2, ZNF460, ZNF135, mimic of miR-320a, lentivirus of ATRX, and their normal controls (NCs) were synthesized by GenePharma. Lipofectamine 2000 (Invitrogen) was used to transfect cells within the study, following by the manufacturer’s protocol.

## Real-time quantitative reverse transcription polymerase chain reaction (qRT-PCR)

To evaluate the expression levels of genes in the current study, a qRT-PCR assay was performed. Briefly, RNA in OSCC cells was isolated using a TRIzol reagent (Invitrogen) following the manufacturer’s instructions. Isolated RNAs were subjected to PrimerScript RT Master Mix (Takara) for cDNA formation according to the manufacturer’s protocol. For miRNA detection, an miRNA qRT-PCR starter kit (Riobo) was used. An ABI Prism 7500 Fast Teal‐Time PCR system was used to perform the PCR experiments. U6 and GAPDH were used as the internal controls. The relative results were analyzed using the 2^−ΔΔCt^ method. The primers used in this study are described below.

**Table:** 

Primers	Forward	Reverse
CircMTO1	5ʹ-GAG CTG TAG AAG ATC TTA TTC-3’	5ʹ-CAC AGG CCA TCC AAG GCA TC-3’
MTO1	5ʹ – TGC ATC AGA GGC TTG GAG AA-3’	5ʹ – AAG GAA GGG GTG ATC TGA CG-3’
miR-320a	5ʹ-TAT TCG CAC TGG ATA CGA CTC CAGC −3’	5ʹ-GTC GTA TCC AGT GCA GGG TCC GAGG −3’
ATRX	5ʹ – GAG ATG TTT CAA GGG AGA GAA GATT – 3’	5ʹ – CCT CCC AAG AGA CAG GTT TCT AA – 3’
U6	5ʹ – CGC TTC ACG AAT TTG CGT GTC AT – 3’	5ʹ – GCT TCG GCA GCA CAT ATA CTA AAAT – 3’
GAPDH	5ʹ-CAC CAT TGG CAA TGA GCG GTTC-3’	5ʹ-AGG TCT TTG CGG ATG TCC ACGT-3’

## RNA fluorescence in situ hybridization (FISH)

To locate the expression of circMTO1 in OSCC cells, we conducted a FISH assay. Cy3-labeled probes for FISH experiments were obtained from RiboBio (Guangzhou, China). A fluorescent in situ hybridization kit (RiboBio Biotechnology) was used to perform the experiment following the manufacturer’s instructions. The results were photographed using a confocal laser microscope (Nikon).

## Western blot

We measured the protein levels of ATRX in treated OSCC cells by conducting western blotting. Total proteins were collected from transfected OSCC cells using radioimmunoprecipitation assay (Beyotime, China). A BCA Protein Assay Kit (Beyotime, China) was used to measure the concentration of the collected proteins following to the manufacturer’s protocol. Proteins were electronically transferred from the gel onto polyvinylidene fluoride (PVDF) membranes (Millipore, Billerica, MA, USA). The members were blocked with 5% skim milk powder for 60 min. The membranes were incubated with the primary antibody (ATRX, Abcam, ab97508, 1:2000) for 12 h, followed by a second antibody (Goat Anti-Rabbit IgG H&L, Abcam, ab150077, 1:500). GAPDH (CST, # 5174S, 1:1000) was used as an internal control. The results were photographed using a gel-imaging system.

## Ethynyl‐2‐deoxyuridine (EdU) incorporation assay

Cell proliferation level was evaluated using the EdU assay, according to the manufacturer’s instructions. Transfected OSCC cells were seeded in 6-well plates (1 × 103 cells for each well) and cultured for 24 h at 37°C in an atmosphere of 5% CO_2_. Next, 10 mmol EdU solution was added to the cells and incubated for 120 min, followed by Hoechst staining for 30 min. The cells were washed with PBS in triplicate. The cells were photographed using a fluorescent microscope. EdU-positive cells were then calculated and analyzed. The experiment was performed in triplicate.

## Transwell assay

The migration and invasion levels of transfected OSCC cells were determined by conducting a transwell assay according to the manufacturer’s protocol. Transwell chambers from Corning Life Sciences were used for the experiments. Matrigel (BD Bioscience) was used for the invasion assay. Cells and medium without FBS were added to the upper chambers, and complete medium was added to the lower chambers. After 48 h, the cells in the upper chambers were stained, photographed, and recorded. The results were analyzed and presented. The experiment was conducted three times.

## RNA binding protein immunoprecipitation (RIP)

To evaluate the potential binding regions of the MTO1 promoter to ZNF460, we performed an RIP assay. In summary, a Magna RIP RNA binding protein immunoprecipitation Kit (Millipore, USA) was used to perform the RIP experiment following the manufacturer’s instructions. Cells were transfected with miR-320a mimic or NC mimic and were cultured for 2 days. Next, the cells were lysed in RIP lysis buffer. Magnetic beads were conjugated with anti-AGO2 antibody (Cell Signaling) or anti-IgG antibody (Cell Signaling) and were subjected to RIP immunoprecipitation buffer to incubate cell isolations. Then, the RNA immunoprecipitation bounds were purified, and the results were analyzed using qRT-PCR assay. The experiment was performed three times.

## RNA pull-down

An RNA pull-down assay was performed to detect putative miRNA targets for circMTO1 and mRNA targets of miR-320 in HOK cells. Biotinylated probes were commercially obtained from RiboBio company (Guangzhou, China). Then, the lysates of CAL-27 and HSC-3 cells were collected and incubated with the biotinylated probes at 4°C overnight on a rotator, followed by treatment with streptavidin-coupled dynabeads (Invitrogen) for 2 h. The RNA-bead mixture was washed five times with a wash buffer. RNA was eluted for qRT-PCR analysis of the enrichment of the indicated RNAs.

## Chromatin immunoprecipitation (ChIP)

To evaluate the occupation of ZNF460 on the MTO1 promoter, a ChIP assay was conducted using the EpiQuik Chromatin Immunoprecipitation Kit (Epigentek, NY, USA) according to the standard protocol. Briefly, cell lysates of cells were collected and treated with 1% formaldehyde for 10 min, followed by quenching with glycine. Then, the lysates were sonicated, digested, and incubated with ChIP-grade ZNF460 antibody (#ab3697, Abcam, Cambridge, UK) and negative control IgG at 4°C overnight. The next day, protein A agarose was added to the above lysates and incubated for one hour. Finally, the ZNF460-bound fragments were eluted for PCR analysis.

## Luciferase reporter assay

To confirm the relationship between the ZNF460/circMTO1/miR-320a/ATRX axis, we used a luciferase reporter gene assay. First, to evaluate the effect of ZNF460 on the activity of the MTO1 promoter sequence, a pGL3-basic vector (Promega, USA) was used to contain the full-length sequence of the MTO1 promoter and co-transfected with siRNA targeting ZNF460 into cells. To assess the interaction between miR-320a and circMTO1 or ATRX, a pSI-check2 vector (Promega, USA) was used to harbor the full-length sequence of the circMTO1 or ATRX promoter and co-transfected with miR-320a mimics into cells. Two days later, a luciferase reporter gene system (Promega, USA) was used to measure the luciferase activity based on the manufacturer’s protocol. Transfections were conducted using a Lipofectamine 3000 Kit (Invitrogen) according to the manufacturer’s instructions.

## Statistical analysis

The results of the experiments were analyzed using the SPSS 10.0. Data are presented as mean ± standard deviation (SD). All experiments were repeated three times. Comparisons among multiple groups were performed using one-way ANOVA. Differences between the groups were calculated using two-tailed t-tests. Statistical significance was set at P < 0.05.

## Results

Here, we hypothesized that circMTO1 might participate in the progression of OSCC. Our results suggest that circMTO1 expression was upregulated in OSCC tissues and samples. The underlying mechanisms investigation showed that circMTO1 promoted ATRX expression in OSCC cells though sponging miR-320a. Furthermore, our results indicated that circMTO1 expression in OSCC cells could be transcriptionally regulated by ZNF460. Our study identified a novel ZNF460/circMTO1/miR-320a/ATRX axis in OSCC progression.

## Characterization and expression of circMTO1 in oral squamous cell carcinoma (OSCC)

**Figure 1.**
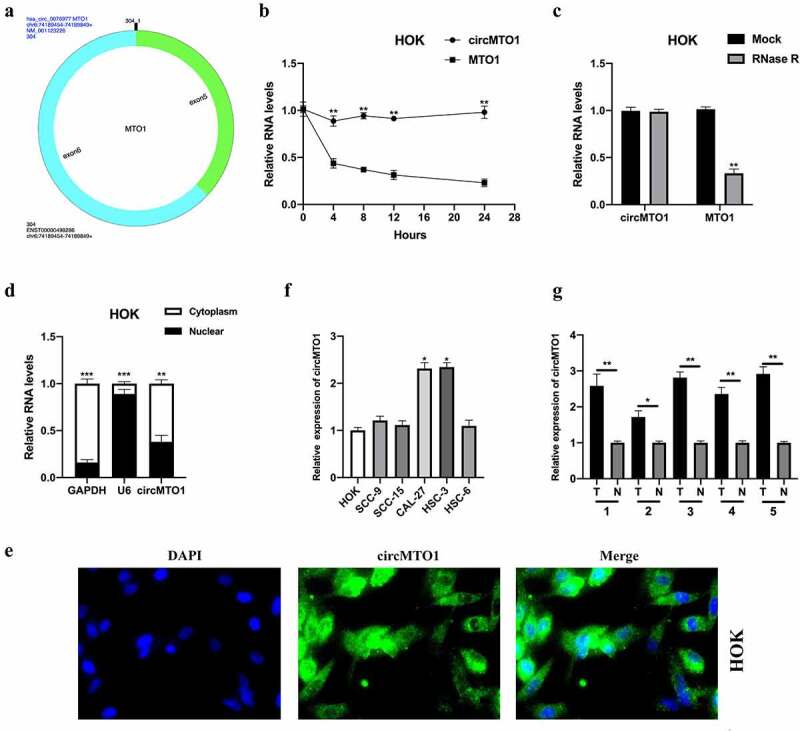
Characterization and expression of circMTO1 in oral squamous cell carcinoma (OSCC). (a) The formation of circMTO1 was presented as a diagram. (b) Measurement of circMTO1 and MTO1 expression in transcription inhibitor actinomycin D-treated HOK cells. (c) Measurement of circMTO1 and MTO1 expression in RNase R administrated HOK cells. (d-e) Cellular RNA fractionation (d) and RNA-FISH (e) assays were conducted to assess the cellular distribution of circMTO1. (f) Measurement of circMTO1 expression in OSCC cell lines. (g) Measurement of circMTO1 expression in five cases of OSCC tumor tissues and their adjacent normal tissues. *P < 0.05, **P < 0.01, ***P < 0.001

CircMTO1 was derived from the MTO1 gene (chr6: 73,461,737–73,509,236), and was back-spliced generated by its five and six exons. The conjunction sites of circMTO1 were shown using CircInteractome tools (https://circinteractome.nia.nih.gov/) (Figure 1a). First, we treated HOK cells with the transcription inhibitor, actinomycin D, to assess the stability of circMTO1. As shown in Figure 1b, the transcript half-life of circMTO1 was obviously longer than that of its linear form. Next, the expression level of circMTO1 and its linear form MTO1 in HOK cells upon RNase R treatment was determined. The circular form (circMTO1), but not its linear form, resisted RNase R treatment (Figure 1c). Cellular RNA fractionation and RNA-FISH assays showed that circMTO1 was mainly located in the cell cytoplasm (Figure 1d-e), suggesting that circMTO1 may have biological functions. Subsequently, we found that circMTO1 was upregulated in OSCC cells (especially in CAL-27 and HSC-3 cells) (Figure 1f) and tissues (Figure 1g). In this section, the characterization and expression of circMTO1 in OSCC cells are partially illustrated.

## Downregulation of circMTO1 inhibited OSCC tumorigenesis

**Figure 2.**
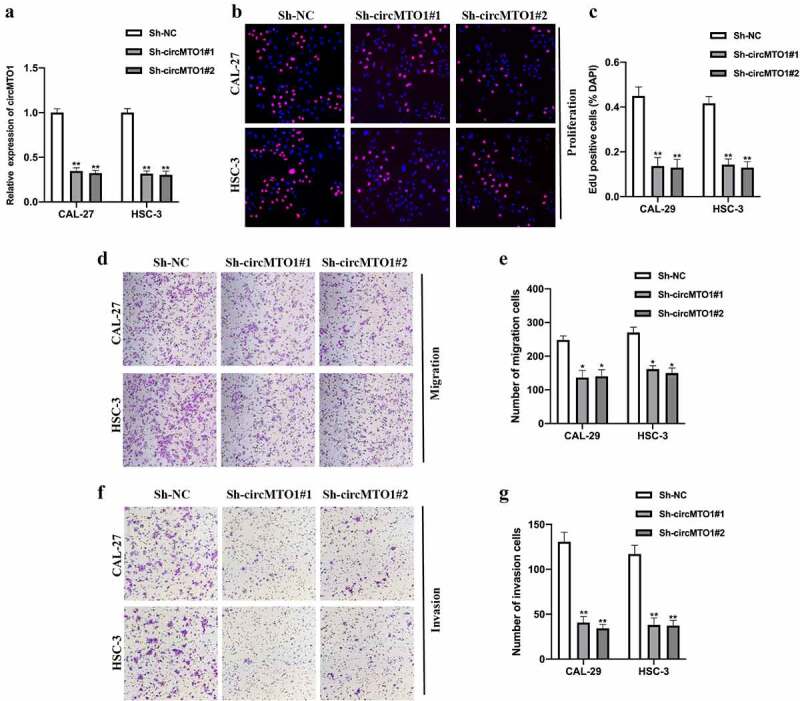
Downregulation of circMTO1 inhibits OSCC tumorigenesis. (a) OSCC cells CAL-27 and HSC-3 were transfected with Sh-NC, Sh-circMTO1#1, and Sh-circMTO1#2 to construct circMTO1 knockdown cell models, and transfection efficiencies were assessed by real-time quantitative reverse transcription polymerase chain reaction (qRT-PCR). (b-c) CircMTO1 knockdown CAL-27 and HSC-3 cells were subjected to EDU assay for proliferation ability detection (b), and statistical analysis was conducted (c). (d-e) Transwell migration assay was performed to evaluate circMTO1 knockdown effect on CAL-27 and HSC-3 cells migration level (d), and the results were analyzed (e). (f-g) Transwell invasion experiment was conducted to measure the invasion level of circMTO1 knockdown CAL-27 and HSC-3 cells (f), and the statistical analysis was presented (g). **P < 0.01

To investigate whether circMTO1 plays its role in OSCC progression, we generated circMTO1 knockdown cell models by transfecting Sh-NC, Sh-circMTO1#1, and Sh-circMTO1#2 into CAL-27 and HSC-3 cells, and the expression level of circMTO1 was measured (Figure 2a). By conducting an EDU assay, we found that circMTO1 knockdown significantly repressed OSCC cell proliferation ability (Figure 2b-c). Moreover, by performing transwell assay, circMTO1 knockdown reduced OSCC cell migration (Figure 2d-e) and invasion (Figure 2f-g) abilities. In this section, we confirmed that circMTO1 is involved in the tumorigenesis progression of OSCC tumorigenesis.

## CircMTO1 directly sponged miR-320a

**Figure 3.**
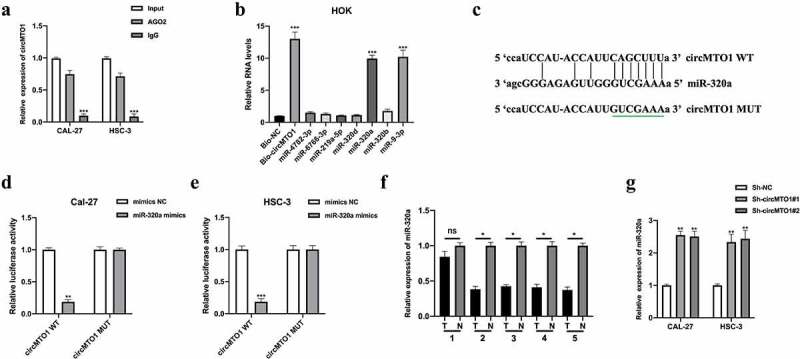
CircMTO1 directly sponges miR-320a. (a) RIP assay using anti-AGO2 and anti-IgG was conducted to assess the miRNA binding possibility of circMTO1, and the results were analyzed by qRT-PCR. (b) Biotinylated RNA pull-down assay was performed to evaluate putative miRNA targets enrichment in circMTO1 probe bounds, and qRT-PCR was used to measure RNAs expression. (c) Wild type (WT) and mutant type (MUT) binding sequences between circMTO1 and miR-320a. (d-e) Dual luciferase reporter assay was applied to assess the interaction between circMTO1 and miR-320a in CAL-27 (d) and HSC-3 (e) cells. (f) Measurement of miR-320a expression in five cases of OSCC tumor tissues and their adjacent normal tissues. (g) Expression of miR-320a in circMTO1 knockdown CAL-27 and HSC-3 cells. *P < 0.05, **P < 0.01, ***P < 0.001

Previous studies suggested that circRNAs act as miRNA sponges to exert their function in cellular progression [17]. First, we evaluated the miRNA binding ability of circMTO1 by conducting an AGO2-RIP assay in OSCC cells. It was found that circMTO1 was significantly enriched in an anti-AGO2 complex compared with an anti-IgG complex (Figure 3a). Subsequently, bioinformatics analysis predicted seven potential miRNA targets of circMTO1. Using a biotinylated RNA pull-down assay, we found that miR-320a and miR-9-3p were abundantly enriched in the biotinylated probe complex (Figure 3b). It has been reported that circMTO1 sponges miR-9-3p ^14^. In this study, we investigated the relationship between circMTO1 and miR-320a. The predicted binding sites between circMTO1 and miR-320a are shown in Figure 3c. Subsequently, the interaction between circMTO1 and miR-320a in OSCC cells was confirmed by a luciferase reporter gene assay (Figure 3d-e). As shown in Figure 3f, qRT-PCR results showed that miR-320a expression were significantly decreased in 4 of 5 (80%) pairs of OSCC tissues suggesting that other mechanisms, such as epigenetic, might contribute to the expression regulation of miR-320a in OSCC. Furthermore, circMTO1 negatively regulated miR-320a expression in OSCC cells (Figure 3g). However, the function of miR-320a in OSCC has not yet been investigated.

## MiR-320a suppressed OSCC tumorigenesis

**Figure 4.**
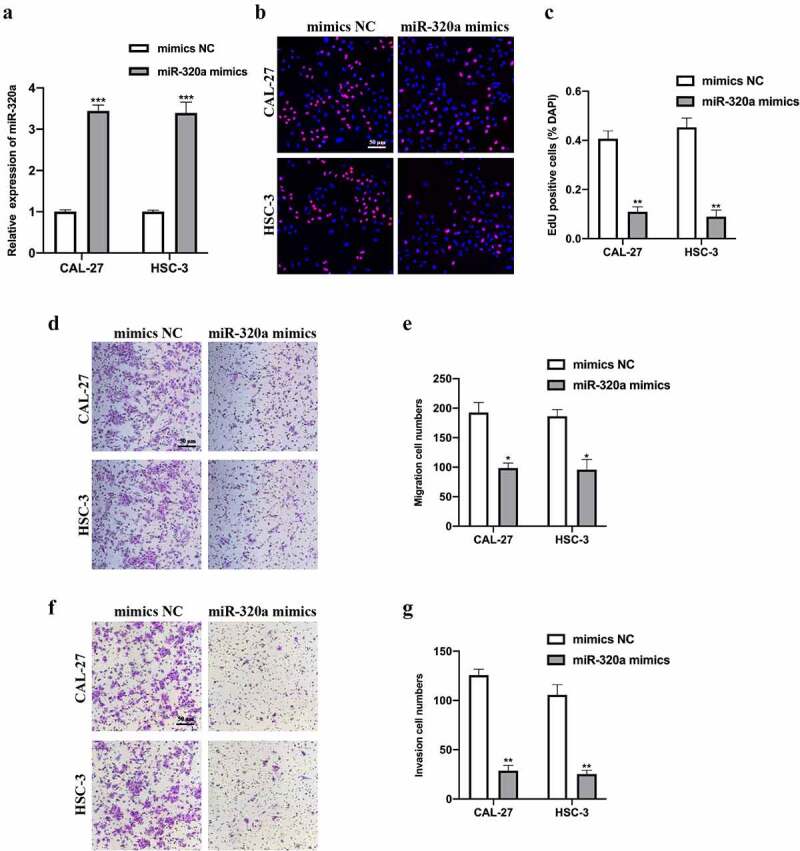
MiR-320a suppresses OSCC tumorigenesis. (a) MiR-320a overexpression cell models were generated by stably transfected NC mimics and miR-320a mimics into CAL-27 and HSC-3 cells, and miR-320a expression was detected by qRT-PCR. (b-c) The proliferation level of miR-320a overexpressed cells were detected by EDU assay (b), and the results were statistically analyzed (c). (d-e) The migration level of miR-320a overexpressed cells were evaluated by transwell assay (d), and the statistical analysis was showed (e). (f-g) The invasion level of miR-320a overexpressed cells was evaluated by transwell assay (f), and the statistical analysis of results was presented (g). *P < 0.05, **P < 0.01, ***P < 0.001

To assess the biological function of miR-320a, we constructed miR-320a overexpression cell models by infecting miR-320a mimics and its NC into CAL-27 and HSC-3 cells. Transfection results were measured (Figure 4a). EDU and transwell assays were performed. EDU assay results indicated that miR-320a overexpression reduced OSCC cell proliferation ability (Figure 4b-c). Transwell migration and invasion assay results suggested that miR-320a overexpression repressed OSCC cell migration (Figure 4d-e) and invasion (Figure 4f-g) level. Overall, our results suggest that miR-320a overexpression suppressed OSCC tumorigenesis progression.

## ATRX was a downstream target of miR-320a

**Figure 5.**
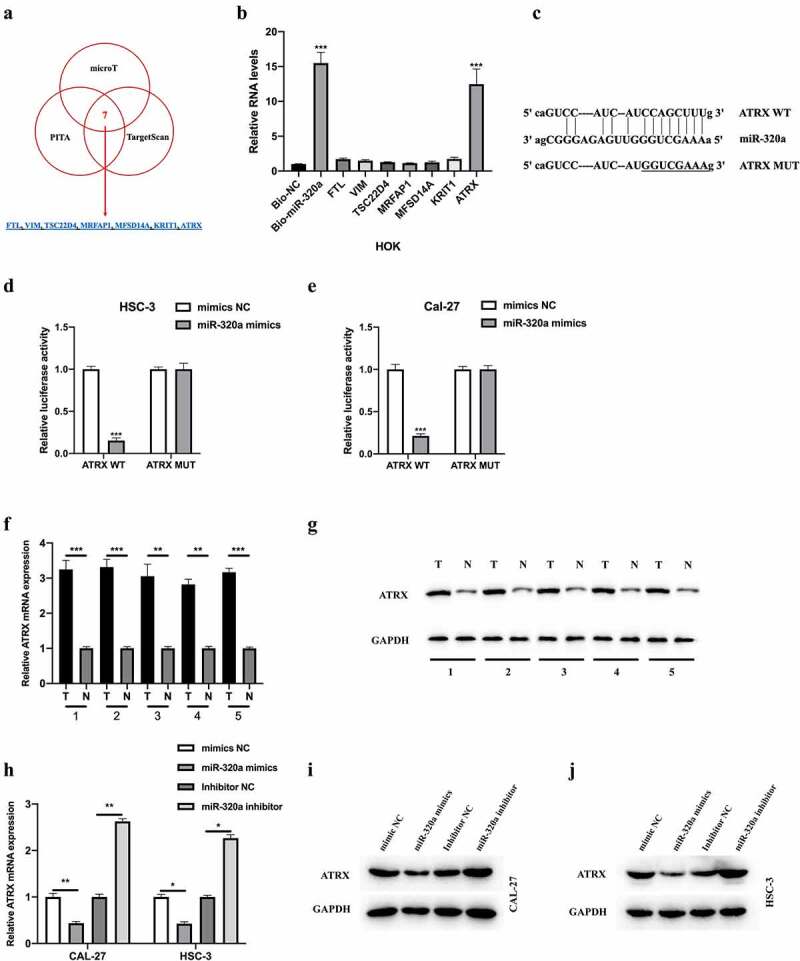
ATRX is a downstream target of miR-320a. The downstream targets of miR-320a were predicted using microT (http://diana.imis.athena-innovation.gr/), PITA (https://genie.weizmann.ac.il/pubs/mir07/mir07_data.html), and Targetscan (http://www.targetscan.org/mamm_31/) with CLIP Data (strict stringency (≥5)), and degradome Data (high stringency (≥3)). (a) Seven putative mRNA targets were showed. (b) Biotinylated RNA pull-down assay was conducted, and qRT-PCR was used to evaluate putative mRNA targets enrichment in biotinylated miR-320a probe bounds. (c) Predicted binding sites between miR-320a and ATRX. (d-e) The interaction between miR-320a and ATRX was assessed by Dual Luciferase Reporter Assay in HSC-3 (d) and CAL-27 (e) cells. (f-g) Measurement of ATRX mRNA (f) and protein (g) expression in five cases of OSCC tumor tissues and their adjacent normal tissues. (h) Expression level of ATRX in CAL-27 and HSC-3 cells upon miR-320a dysregulation were measured by qRT-PCR. (i-j) Expression level of ATRX in miR-320a dysregulated CAL-27 (G) and HSC-3 (h) cells were detected by western blot. *P < 0.05, **P < 0.01, ***P < 0.001

We further explored the downstream targets of miR-320a using bioinformatics analysis. Seven potential targets were selected for examination (Figure 5a). The results of the biotinylated RNA pull-down assay indicated that ATRX might be the target of miR-320a (Figure 5b). The wild-type and mutant-type binding sites between ATRX and miR-320a were synthesized (Figure 5c). A dual-luciferase reporter assay was performed, and the interaction between miR-320a and ATRX in OSCC cells was confirmed (Figure 5d-e). Subsequently, we found that the mRNA (Figure 5f) and protein (Figure 5g) levels of ATRX in OSCC tissues were increased and were negatively regulated by miR-320a in OSCC cells (Figure 5h-g). Collectively, we found that miR-320a targeted ATRX in OSCC cells and negatively regulated its expression.

## CircMTO1 promoted OSCC progression through miR-320a/ATRX

**Figure 6.**
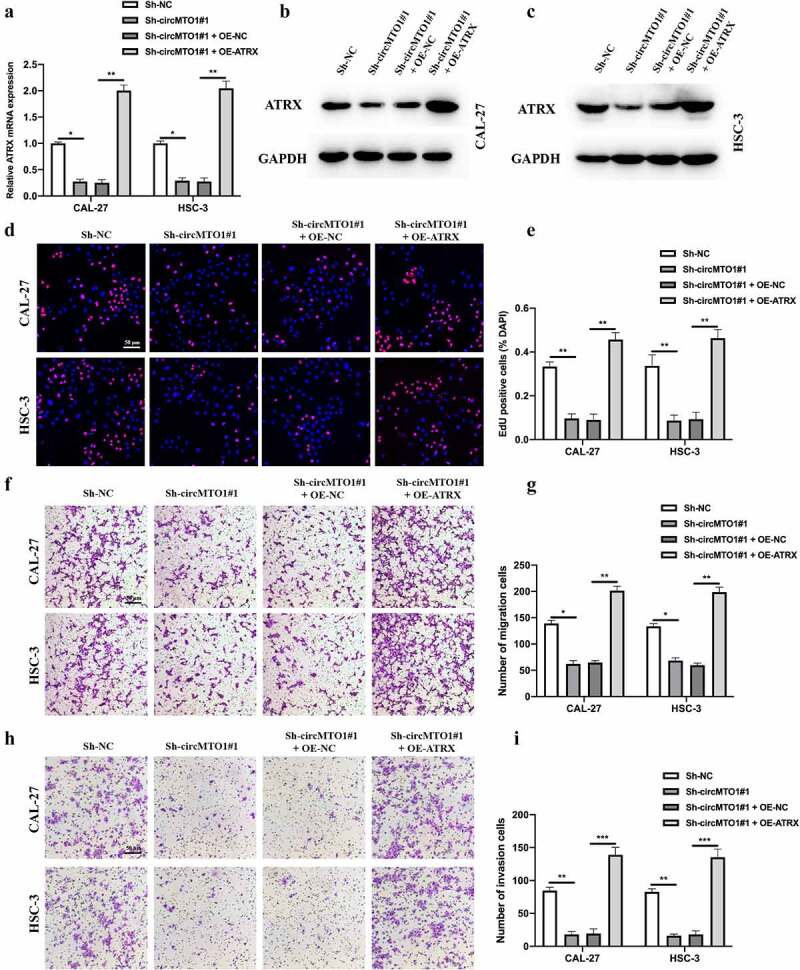
CircMTO1 promotes OSCC progression through miR-320a/ATRX. (a-c) Cell models were constructed by transfecting Sh-NC, Sh-circMTO1#1, Sh-circMTO1#1 + OE-NC, and Sh-circMTO1#1 + OE-ATRX into CAL-27 and HSC-3 cells, and the expression level of ATRX was measured by qRT-PCR (a) and western blot (b and c). (d-e) The proliferation level of treated as indicated cells were measured by EDU assay (d), and statistical analysis results was presented (e). (f-g) The migration level of treated as indicated cells were evaluated by transwell assay (f), and the statistical analysis results is shown (g). (h-i) The invasion level of treated as indicated cells were evaluated by transwell assay (h), and the statistical analysis results was presented (i). *P < 0.05, **P < 0.01, ***P < 0.001

To verify the role of the circMTO1/miR-320a/ATRX axis in OSCC progression. Cell models were constructed, and the expression level of ATRX in treated CAL-27 and HSC-3 cells was measured by qRT-PCR (Figure 6a) and western blotting (Figure 6b-c). EDU and transwell experiments were conducted to evaluate the biological functions of treated as indicated OSCC cells. EDU assay results indicated that the inhibitory effect of circMTO1 knockdown on OSCC cell proliferation could be reversed by ATRX overexpression (Figure 6d-e). Furthermore, we found that ATRX overexpression also rescued the phenomena in which OSCC cell migration (Figure 6f-g) and invasion (Figure 6h-i) abilities were reduced by circMTO1 knockdown. Here, our results have partially demonstrated the function of circMTO1/miR-320a/ATRX in OSCC progression.

## CircMTO1 was transcriptionally upregulated by ZNF460

**Figure 7.**
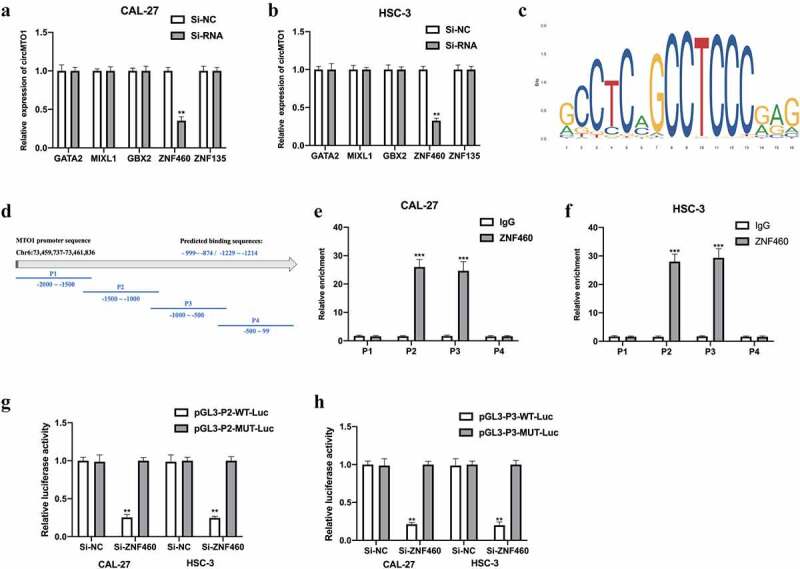
CircMTO1 is transcriptionally upregulated by ZNF460. Transcription factors of circMTO1 were predicted using NCBI (https://www.ncbi.nlm.nih.gov/), UCSC (http://genome.ucsc.edu/), and JASPAR (http://jaspar.genereg.net/) datasets. Five potential factors were predicted (GATA2, MIXL2, GBX2, ZNF460, and ZNF135). (a-b) Expression level of circMTO1 in CAL-27 (a) and HSC-3 (b) cells pre-treated with indicated SiRNAs were measured by qRT-PCR. (c) The predicted binding sites of ZNF460. (d) The promoter of circMTO1 was presented, and the promoter was spliced into P1-P4 section. (e-f) ChIP assay was used in CAL-27 (e) and HSC-3 (f) cells, and the results were analyzed using qRT-PCR. G-(h) CAL-27 (g) and HSC-3 (h) cells were co-transfected with indicated luciferase reporter vectors and siRNAs; luciferase activities were measured. *P < 0.05, **P < 0.01, ***P < 0.001

CircRNAs have been reported to be regulated by transcription factors [18]. In this study, we further investigated the upstream regulators of circMTO1. The NCBI, UCSC, and JASPAR datasets were used in this study. Five potential transcription regulators were selected for this study. We found that in the CAL-27 cells (Figure 7a) and HSC-3 cells (Figure 7b), circMTO1 expression was significantly decreased upon Si-ZNF460 transfection, suggesting that ZNF460 might regulate circMTO1 expression. The predicted binding sites between ZNF460 (Figure 7c) and the MTO1 promoter the MTO1 promoter are shown in Figure 7c and Figure 7d, respectively. The predicted binding regions (P2 and P3) were verified by ChIP-RNA assay (Figure 7e-f) and confirmed by luciferase reporter gene assay (Figure 7g-h). Overall, our results suggest that circMTO1 expression in OSCC cells was transcriptionally regulated by ZNF460.

## Discussion

OSCC is the most malignant tumor of oral cancers and the 11^th^ most common cancer type worldwide. The 5-year survival rate of OSCC only remains nearly 50%, and approximately 145,000 mortalities were caused by OSCC [19,20]. Although multiple clinical interventions, including surgery, radiotherapy, and chemotherapy, have been successfully applied to patients with OSCC, the 5-year survival rate of OSCC patients with OSCC has not been significantly improved [21,22]. The complex pathogenesis provides an accurate explanation for OSCC initiation and progression.

First, our study elucidated the circRNA features and expression levels of circMTO1 in OSCC tissues and cells; circMTO1 was highly expressed both in OSCC tissues and cells. Next, we found that circMTO1 knockdown repressed OSCC cell proliferation, migration, and invasion level. These results suggest that circMTO1 plays a functional role in OSCC progression. CircRNA serves as a molecular sponge of miRNA and modulates the downstream mRNA target of miRNA, which is a classical network in various pathogenesis [23–27]. In OSCC, for instance, circ_0000140 attenuates OSCC progression by sponging miR-31 to promote LATS2 expression [28]. Hsa_circRNA_100533 inhibits OSCC development through regulating GNAS by targeting hsa_miR_933 [29]. CircBICD2 regulates OSCC progression by targeting miR-149-5p/IGF2BP1 [30]. Based on the crucial role of the circRNA/miRNA/mRNA network in OSCC development, we performed bioinformatics analysis and a serial experiment to demonstrate the underlying mechanisms of circMTO1. Our results suggest that cricMTO1 promotes ATRX expression by sponging miR-320a in OSCC cells.

The expression characteristics and biological functions of miR-320a have been well elucidated in many cancers, including cervical cancer, non-small cell lung cancer, cholangiocarcinoma [31–33]. However, whether miR-320a regulates OSCC initiation or progression remains unknown. Our study found that miR-320a downregulation repressed OSCC cell proliferation, migration, and invasion abilities. Furthermore, miR-320a targeted ATRX in OSCC cells, and neither miR-320a nor ATRX was investigated in OSCC. Our study partially revealed that circMTO1 regulates OSCC development by regulating ATRX expression by sponging miR-320a. We also investigated the upstream mechanism of circMTO1. ZNF460, a transcription regulator, induced circMTO1 expression in OSCC cells.

Nevertheless, our study demonstrated the functional role of the ZNF460/circMTO1/miR-320a/ATRX pathway in OSCC cells. Further investigation is required in our study. First, sufficient clinical specimens of OSCC are needed to demonstrate the clinical significance of the ZNF460/circMTO1/miR-320a/ATRX pathway. Next, the relationship between the ZNF460/circMTO1/miR-320a/ATRX pathway and OSCC cellular behaviors requires in-depth study.

## Conclusion

In summary, our study elucidated the biological function of circMTO1 in OSCC cells. Subsequently, we demonstrated the downstream and upstream mechanisms of circMTO1. We found that circMTO1 induced by ZNF460 promotes OSCC progression by targeting the miR-320a/ATRX pathway.

## Data Availability

The datasets and materials used in this study are available based on reasonable requested.

## Highlights

- CircMTO1 is upregulated in OSCC tissues and cells.
- CircMTO1 knockdown represses the proliferation, migration, invasion abilities of OSCC cells.
- CircMTO1 modulates ATRX expression though targeting miR-320a.
- CircMTO1 expression in OSCC cells could be transcriptionally regulated by ZNF460.

## References

[1] Choi S, Myers JN. Molecular pathogenesis of oral squamous cell carcinoma: implications for therapy. J Dent Res. 2008;87(1):14–32. 18096889 10.1177/154405910808700104

[2] Siegel RL, Miller KD, Jemal A. Cancer statistics, 2017. CA Cancer J Clin. 2017;67(1):7–30. 28055103 10.3322/caac.21387

[3] Chi AC, Day TA, Neville BW. Oral cavity and oropharyngeal squamous cell carcinoma–an update. CA Cancer J Clin. 2015;65(5):401–421. 26215712 10.3322/caac.21293

[4] Pollaers K, Hinton-Bayre A, Friedland PL, et al. AJCC 8th Edition oral cavity squamous cell carcinoma staging - Is it an improvement on the AJCC 7th Edition? Oral Oncol. 2018;82:23–28. 29909897 10.1016/j.oraloncology.2018.04.018

[5] Ramos-Garcia P, Gonzalez-Moles MA, Gonzalez-Ruiz L, et al. Prognostic and clinicopathological significance of cyclin D1 expression in oral squamous cell carcinoma: a systematic review and meta-analysis. Oral Oncol. 2018;83:96–106. 30098785 10.1016/j.oraloncology.2018.06.007

[6] Lasda E, Parker R. Circular RNAs: diversity of form and function. RNA. 2014;20(12):1829–1842. 25404635 10.1261/rna.047126.114 PMC4238349

[7] Jeck WR, Sorrentino JA, Wang K, et al. Circular RNAs are abundant, conserved, and associated with ALU repeats. RNA. 2013;19(2):141–157. 23249747 10.1261/rna.035667.112 PMC3543092

[8] Kristensen LS, Hansen TB, Veno MT, et al. Circular RNAs in cancer: opportunities and challenges in the field. Oncogene. 2018;37(5):555–565. 28991235 10.1038/onc.2017.361 PMC5799710

[9] Saikishore R, Velmurugan P, Ranjithkumar D, et al. The circular RNA-miRNA axis: a special RNA signature regulatory transcriptome as a potential biomarker for OSCC. Mol Ther Nucleic Acids. 2020;22:352–361. 33230440 10.1016/j.omtn.2020.09.001 PMC7530261

[10] Fan HY, Jiang J, Tang YJ, et al. CircRNAs: a new chapter in oral squamous cell carcinoma biology. Onco Targets Ther. 2020;13:9071–9083. 32982296 10.2147/OTT.S263655 PMC7494394

[11] Zhao W, Cui Y, Liu L, et al. Splicing factor derived circular RNA circUHRF1 accelerates oral squamous cell carcinoma tumorigenesis via feedback loop. Cell Death Differ. 2020;27(3):919–933. 31570856 10.1038/s41418-019-0423-5 PMC7206121

[12] Liu J, Jiang X, Zou A, et al. circIGHG-induced epithelial-to-mesenchymal transition promotes oral squamous cell carcinoma progression via miR-142-5p/IGF2BP3 signaling. Cancer Res. 2021;81(2):344–355. 33203701 10.1158/0008-5472.CAN-20-0554

[13] Gao L, Dou ZC, Ren WH, et al. CircCDR1as upregulates autophagy under hypoxia to promote tumor cell survival via AKT/ERK(1/2)/mTOR signaling pathways in oral squamous cell carcinomas. Cell Death Dis. 2019;10(10):745. 31582727 10.1038/s41419-019-1971-9 PMC6776509

[14] Han D, Li J, Wang H, et al. Circular RNA circMTO1 acts as the sponge of microRNA-9 to suppress hepatocellular carcinoma progression. Hepatology. 2017;66(4):1151–1164. 28520103 10.1002/hep.29270

[15] Chen M, Ai G, Zhou J, et al. circMTO1 promotes tumorigenesis and chemoresistance of cervical cancer via regulating miR-6893. Biomed Pharmacother. 2019;117:109064. 31226633 10.1016/j.biopha.2019.109064

[16] Li Y, Wan B, Liu L, et al. Circular RNA circMTO1 suppresses bladder cancer metastasis by sponging miR-221 and inhibiting epithelial-to-mesenchymal transition. Biochem Biophys Res Commun. 2019;508(4):991–996. 30551873 10.1016/j.bbrc.2018.12.046

[17] Bose R, Ain R. Regulation of transcription by circular RNAs. Adv Exp Med Biol. 2018;1087:81–94. 30259359 10.1007/978-981-13-1426-1_7

[18] Kristensen LS, Andersen MS, Stagsted LVW, et al. The biogenesis, biology and characterization of circular RNAs. Nat Rev Genet. 2019;20(11):675–691. 31395983 10.1038/s41576-019-0158-7

[19] Coleman MP, Quaresma M, Berrino F, et al. Cancer survival in five continents: a worldwide population-based study (Concord). Lancet Oncol. 2008;9(8):730–756. 18639491 10.1016/S1470-2045(08)70179-7

[20] Warnakulasuriya S. Global epidemiology of oral and oropharyngeal cancer. Oral Oncol. 2009;45(4–5):309–316. 18804401 10.1016/j.oraloncology.2008.06.002

[21] Krstevska V. Evolution of treatment and high-risk features in resectable locally advanced Head and Neck squamous cell carcinoma with special reference to extracapsular extension of nodal disease. J BUON. 2015;20:943–953. 26416042

[22] Pring M, Prime S, Parkinson EK, et al. Dysregulated TGF-beta1-induced Smad signalling occurs as a result of defects in multiple components of the TGF-beta signalling pathway in human head and neck carcinoma cell lines. Int J Oncol. 2006;28:1279–1285. 16596245

[23] Liang ZZ, Guo C, Zou MM, et al. circRNA-miRNA-mRNA regulatory network in human lung cancer: an update. Cancer Cell Int. 2020;20(1):173. 32467668 10.1186/s12935-020-01245-4 PMC7236303

[24] Han TS, Hur K, Cho HS, et al. Epigenetic associations between lncRNA/circRNA and miRNA in hepatocellular carcinoma. Cancers (Basel). 2020;12(9):2622. 10.3390/cancers12092622 PMC7565033 32937886

[25] Li M, Duan L, Li Y, et al. Long noncoding RNA/circular noncoding RNA-miRNA-mRNA axes in cardiovascular diseases. Life Sci. 2019;233:116440. 31047893 10.1016/j.lfs.2019.04.066

[26] Meng S, Zhou H, Feng Z, et al. CircRNA: functions and properties of a novel potential biomarker for cancer. Mol Cancer. 2017;16(1):94. 28535767 10.1186/s12943-017-0663-2 PMC5440908

[27] Shang Q, Yang Z, Jia R, et al. The novel roles of circRNAs in human cancer. Mol Cancer. 2019;18(1):6. 30626395 10.1186/s12943-018-0934-6 PMC6325800

[28] Peng QS, Cheng YN, Zhang WB, et al. circRNA_0000140 suppresses oral squamous cell carcinoma growth and metastasis by targeting miR-31 to inhibit Hippo signaling pathway. Cell Death Dis. 2020;11(2):112. 32041942 10.1038/s41419-020-2273-y PMC7010827

[29] Zhu X, Shao P, Tang Y, et al. hsa_circRNA_100533 regulates GNAS by sponging hsa_miR_933 to prevent oral squamous cell carcinoma. J Cell Biochem. 2019;120(11):19159–19171. 31297884 10.1002/jcb.29245

[30] Qiu L, Zheng L, Gan C, et al. circBICD2 targets miR-149-5p/IGF2BP1 axis to regulate oral squamous cell carcinoma progression. J Oral Pathol Med. 2021;50(7):668–680. 33382158 10.1111/jop.13156

[31] Hong H, Zhu H, Zhao S, et al. The novel circCLK3/miR-320a/FoxM1 axis promotes cervical cancer progression. Cell Death Dis. 2019;10(12):950. 31831728 10.1038/s41419-019-2183-z PMC6908646

[32] Lu M, Hu C, Wu F, et al. MiR-320a is associated with cisplatin resistance in lung adenocarcinoma and its clinical value in non-small cell lung cancer: a comprehensive analysis based on microarray data. Lung Cancer. 2020;147:193–197. 32731058 10.1016/j.lungcan.2020.06.020

[33] Zhu H, Zhai B, He C, et al. LncRNA TTN-AS1 promotes the progression of cholangiocarcinoma via the miR-320a/neuropilin-1 axis. Cell Death Dis. 2020;11(8):637. 32801339 10.1038/s41419-020-02896-x PMC7429853

